# Facts Relating to the Period at Which Menstruation Ceases in Woman, and the Effects Thereby Produced on the Health

**Published:** 1844-11-30

**Authors:** 


### FACTS RELATING TO THE PERIOD AT WHICH MENSTRUATION CEASES IN WOMAN, AND THE EFFECTS THEREBY PRODUCED ON THE HEALTH.

BY MM. DE MEYNCK AND KLUYSKENS.

It is a fact that much more attention has been directed to the period at which the catamenia appear, than to that at which they cease; one cause of which, probably, may be the greater number of females subject to the former of these phenomena, and their tender age, and the little importance, in the majority of cases attached to the latter. Too little attention, indeed, has probably been given to the cessation of the catamenia, and many states have been attributed to a morbid condition of the uterus, which were in reality nothing more than a prolongation of the normal state, and vice versa. The following facts may, perhaps, throw light on the subject.

*Return of the menses at an advanced age, cure of some obstinate affections.*

Case 1. A nun, aged 62, of a sanguineous constitution, was seized with obstinate gastralgia, after the cessation of the menses at the age of 52. For nine years she was treated by many different medical men without relief. The following are the symptoms from which she suffered when I saw her. Palpitation, with pain at the epigastrium two or three hours after taking food; digestion slow and difficult; fetid eructations, constipation, no appetite, tongue clean, urine watery, pulse natural, character irritable, sleep good. Light tonic regimen, occasional application of leeches to the anus, keeping up the spirits (moral) of the patient. In the course of a short time her sufferings were alleviated, the constipation was less obstinate, and she experienced a feeling of comfort unknown for many years. The melioration continued up to the period when a sanguineous evacuation took place from the uterus; it lasted four days, was unaccompained with pain or any functional disturbance whatever, but there was considerable amendment in all the gastralgic phenomena. Another discharge occurred twenty-eight days after the first, and from that period the menses have recurred with their normal regularity, and now, though the lady is aged 73, they return with as great regularity as they did when she was at the age of 20. Since this re-appearance of the menses, not only have all the gastric symptoms disappeared, but she has continued to enjoy the most perfect health.

Case 2. A nun, aged 90, had menstruated regularly from the age of 15 to 52; at this latter period she was seized with violent colic, which continued to recur at intervals for the space of two years. To this succeeded tic douloureux in well marked paroxysms; it resisted a variety of remedies, and ceased only at the age of 60, at which period the menses re-appeared without pain, and they have recurred regularly since every month; she enjoys excellent health, and is in full possession of all her faculties, moral and intellectual; she exhibits, besides, tastes and ideas which are generally confined to youth.

Examples of menstruation continuing to an advanced age, are not uncommon. Many authors mention females who have given birth to healthy children, at the advanced age of 67 and 70 (?) the two cases cited above are opposed to the opinion commonly entertained, that prolonged menstruation is generally met with in females addicted to sexual pleasures. The subjects of the two preceding cases were devoted to celibacy, and led a rigid and austere life.

*Supplementary hemorrhage from the mamma at an advanced age, alternating with paroxysms of asthma, and terminating in a cancer of the breast.*

Case 3. M., of a sanguineous constitution, has had three children, and for 40 years enjoyed excellent health. At that age the menses becoming less abundant, she began to experience wandering pains in both extremities, with a feeling of oppression and fatigue. This state continued two years without exciting attention. At the end of that period, however, the symptoms increased, and she was seized with occasional attacks of asthma, which usually preceded the appearance of the menses; at each return of these the left breast became swollen, with an intolerable feeling of itchiness around the nipple, the other breast remained perfectly healthy; the swelling of the breast sensibly increased; the veins became prominent, and the nipple in a state of erythism, and surrounded with an areola of a violet red colour.

The menses now became more and more scanty, and the attacks of asthma, more frequent and intense, resisted all the means employed. At this time a small quantity of pale blood flowed from the enlarged mamma, it escaped in drops, and might weigh 20 or 30 grs., she was then 52, and the menses had entirely disappeared.

This slight hemorrhage completely relieved the asthmatic attacks, and the breast regained its natural size and color. From this period, a state of orgasm occurred periodically in the breast, similar to what is observed in the uterus previous to the appearance of the menses. The discharge of blood followed, and when this was scanty, the attacks of asthma recurred with greaty intensity.

She lived for five years and a half with this singular menstural flux, but at that period a scirrhous tumour appeared in the breast, which soon passed into the state of cancer, under which she sunk, and died at the age of 58.—*Ib.*, from *Gazette Med.*, Sept. 1844.

